# Designing sustainable robotic surgery for NHS scale-up: direct electricity measurement and an implementation-ready energy mitigation bundle in colorectal cancer resections

**DOI:** 10.1308/rcsann.2026.0065

**Published:** 2026-07-02

**Authors:** S Lodhia, V Pegna, T Rockall

**Affiliations:** Royal Surrey County Hospital, UK

**Keywords:** Robotic-assisted surgery, Laparoscopic surgery, Colorectal cancer, Energy consumption, Sustainability

## Abstract

**Introduction:**

Robotic-assisted surgery is expanding across specialties due to advantages including instrument dexterity, depth perception and improved ergonomics. NHS England projections indicate rapid scale-up of robotic surgery to ∼500,000 robot-supported operations annually by 2035, with robotic assistance becoming the default for ∼90% of keyhole procedures. Operating theatres are a key focus for decarbonisation, and equipment energy demand represents an actionable lever. This study quantified electricity consumption for robotic versus laparoscopic cancer surgery, translating to an implementation-ready mitigation bundle to support robotic programme expansion while minimising avoidable energy use.

**Methods:**

Electricity consumption on robotic and laparoscopic equipment was measured during colorectal cancer resections, capturing standby during setup/turnover, in-use during operating and out-of-hours standby. Readings were obtained during defined operating states, then modelled using prespecified setup, operative and clear-away durations based on local theatre workflow observations and published evidence of longer operative times associated with robotic colorectal surgery. Energy consumption, costs and annualised projections were calculated.

**Results:**

Robotic equipment drew substantially higher power than laparoscopic stacks in both standby and in-use states. Robotic cases used over six times more electricity per operation than laparoscopic cases, driven by longer operative duration and higher system power. Out-of-hours standby for robotic components contributed materially to annual electricity use and cost.

**Conclusions:**

Alongside clinical outcomes and productivity metrics, energy use and carbon implications should be incorporated into robotic surgery programme governance. Mitigations include routine measurement, standby power management within manufacturer constraints, theatre workflow optimisation and procurement standards aligned to sustainable operating theatre guidance.

## Introduction

The adoption of minimally invasive surgery has transformed operative care. In 1985, Erich Mühe performed the first laparoscopic cholecystectomy – a milestone that preceded widespread acceptance of laparoscopic approaches.^1^ Robotic-assisted surgery emerged later, with early robotic cholecystectomy experience reported in the 1990s and subsequent expansion across specialties.^2,3^ Robotic platforms are now deployed in multiple fields including urology, general surgery and gynaecology.^4,5^

Robotic systems are often a different means of delivering similar minimally invasive procedures to laparoscopy. Their dissemination has been driven by perceived benefits including improved dexterity, enhanced visualisation and surgeon ergonomics, with growing attention to training structures and curricula for robotic practice.^5–7^ In colorectal cancer surgery, robotic rectal cancer approaches have been associated with favourable outcomes compared with laparoscopy in large retrospective cohorts, although evidence varies by procedure type and study design.^5^

However, healthcare is now required to deliver innovation under explicit sustainability constraints. The NHS has committed to net zero by 2040 for emissions it controls directly and by 2045 for emissions it can influence, including supply chain impacts.^8^ Operating theatres are recognised as major contributors to hospital environmental impact and a focus for quality improvement and decarbonisation initiatives.^9,10^ A key theatre-based opportunity is avoidable electricity use, including out-of-hours standby energy, where equipment may remain powered unnecessarily overnight and at weekends.^10–13^

### Why laparoscopy is the comparator (and why this comparison matters now)

Laparoscopy remains the predominant minimally invasive approach in many colorectal pathways and is the modality around which many theatre sustainability efforts are currently being designed and implemented.^13^ Robotic surgery, by contrast, is forecast to grow rapidly. NHS England projections indicate around 500,000 robot-supported operations per year by 2035 (from ∼70,000 in 2023-2024), and robotic assistance for around nine in ten keyhole procedures over the same period.^12,13^ Therefore, understanding the incremental electricity demand of robotics relative to laparoscopy is essential for future planning: without mitigation, scaling robotic capacity risks scaling avoidable energy consumption, emissions and cost.

In addition, some robotic systems have manufacturer guidance that certain components should remain connected to mains power to maintain battery readiness, which can create persistent standby demand outside working hours.^14^ This study directly measures electricity consumption for robotic and laparoscopic colorectal cancer surgery equipment to quantify (i) standby and in-use power demand, (ii) per-case electricity use and cost and (iii) out-of-hours standby energy and annualised projections. We then translate these findings into a concise mitigation bundle that can be adopted in robotic programme governance as services expand.

## Methods

### Study setting and procedures

We focused on colorectal cancer resections because sufficient volumes of both laparoscopic and robotic cases were undertaken. Procedures included anterior resections and right hemicolectomies. Robotic and laparoscopic cases were selected from the same colorectal cancer service and represented comparable minimally invasive colorectal resections performed in the same theatre environment. However, this was not a matched clinical outcomes study and cases were not matched for patient factors, tumour location, complexity, surgeon or case-mix. The purpose of the comparison was therefore to quantify modality-specific equipment electricity use under realistic local operating conditions, rather than to compare clinical outcomes between equivalent patient cohorts.

### Equipment included/excluded

We measured electricity demand for equipment specific to each modality:

- Laparoscopic stack, including light source, insufflator and monitors;
- Robotic system (da Vinci Xi), including mobile/patient cart, imaging cart and surgeon console(s).

We excluded equipment common to both approaches (e.g., anaesthetic machine, theatre lighting, general computers, shared screens) to isolate incremental electricity attributable to modality choice.

### Measurement approach

Power monitors were applied to equipment power supplies. We recorded:

- Standby: powered on during setup/turnover
- In-use: active operation
- Out-of-hours standby: overnight and weekends

For each modality, power draw was measured directly from the equipment power supply during each defined state. Standby readings were taken when the relevant stack or robotic system was powered on but not being used actively for surgery. In-use readings were taken once the equipment was active during the operative phase. Out-of-hours standby readings were taken during overnight and weekend periods when equipment remained connected to mains power. Readings were recorded in watts and repeated five times for each state, with the recorded values used to calculate modelled energy consumption in kWh. Per-case energy consumption was then calculated by multiplying measured power draw by the assumed duration of each operating state.

The modelled durations were chosen to reflect local theatre workflow observations for colorectal cancer resections and were cross-checked against published evidence showing longer operative times for robotic colorectal surgery compared with laparoscopic surgery, as well as published data on additional robotic preparation, draping and docking time. Measurements were repeated five times, and modelled per-case energy consumption was calculated using typical setup/clear-down and operative durations.

### Costs and annual extrapolation

Electricity tariffs were obtained from the Trust Estates Department: £0.14/kWh (day) and £0.11/kWh (night/weekend). The Trust’s annual electricity consumption and cost were recorded for contextualisation. Out-of-hours standby energy was extrapolated to annual values per robot and for a three-robot estate.

## Results

### Theatre equipment and operative durations

Standard theatre equipment included:

- Laparoscopic stack;
- Robotic system: mobile/patient cart, imaging cart, surgeon console and second console.

Modelled durations:

- Laparoscopic: two hours operative + 30 minutes setup + 30 minutes clear-away;
- Robotic: four hours operative + 45 minutes setup + 45 minutes clear-away.

These timings were used as pragmatic modelling assumptions rather than measured case-specific operative times. They were based on local theatre workflow observations for colorectal cancer resections and reflect the additional preparation, docking, undocking and clear-away requirements associated with robotic surgery. They are also consistent with published comparative colorectal surgery literature reporting longer operative times for robotic cases compared with laparoscopic cases, including recent systematic review evidence and cohort data showing a longer median or mean operative duration for robotic colorectal surgery. Published work on da Vinci Xi workflow also supports the inclusion of additional draping and docking time when modelling robotic theatre energy use.^15^

### Robotic component power

The mobile/patient cart accounted for the largest proportion of both standby and in-use power and is the component typically maintained on standby out-of-hours (Table 1).^16^

**Table 1:** Robotic component power draw

Component	Standby (W)	In-use (W)
Mobile/patient cart	158.5	732.25
Imaging cart	39.1	120.4
Consoles (×2)	137.8	410
Robot total	335.4	1,262.65

### Robotic versus laparoscopic power draw

Robotic power draw was approximately threefold higher than laparoscopy in both standby and in-use states (Table 2).

**Table 2:** Comparative power draw

State	Laparoscopic (W)	Robotic (W)
Standby	122.5	335.4
In-use	396.8	1,262.65

### Energy per operation

Robotic cases used over six times the electricity per operation (Table 3).

**Table 3:** Energy per operation

Modality	kWh per operation
Laparoscopic	54.97
Robotic	333.22

### Electricity cost per operation

Table 4 shows the electricity per operation.

**Table 4:** Electricity cost per operation

Modality	Cost per operation (£)
Laparoscopic	7.14
Robotic	43.32

### Out-of-hours standby and annualised projections

Laparoscopic equipment was powered down out of hours, incurring no out-of-hours electricity use in this setting. Robotic systems required ongoing standby power for components maintained ready for use (Tables 5 and 6).^14^

**Table 5:** Annualised out-of-hours electricity

Period	Robot (kWh)	Total per year (3 robots, kWh)
Overnight (6pm–8am)	9.51	—
Weekend (Friday 6pm–Monday 8am)	589.62	—
Year (per robot)	58,353.36	175,060.08

**Table 6:** Annualised out-of-hours electricity cost

Period	Robot (£)	3 robots per year (£)
Overnight	14.65	—
Weekend	64.86	—
Year (per robot)	6,418.87	19,256.61

## Discussion

This study demonstrates that robotic-assisted colorectal cancer resections consume substantially more electricity than laparoscopic cases in our measurements. The clearest interpretation is that robotic equipment has higher electricity demand than laparoscopic equipment in both standby and in-use states, and that this relative difference may become important when robotic surgery is scaled across high-volume surgical programmes. The difference reflects: (i) higher system power demand in both standby and in-use states, (ii) longer operative duration and (iii) nontrivial out-of-hours standby demand for robotic components maintained ready for use. Manufacturer guidance to keep certain robotic components connected to mains power to maintain battery readiness is a plausible driver of persistent standby consumption.^14^

The NHS is actively scaling robotics. NHS England projections indicate approximately 500,000 robot-supported operations per year by 2035, and robotic assistance as the default for around 90% of keyhole procedures.^11,12^ This means any incremental electricity demand will scale with national implementation. Although the direct electricity cost per individual case may be modest relative to total operative cost, the cumulative effect may become material across a high-volume robotic programme, particularly where equipment remains powered during avoidable standby. Without explicit mitigation strategies, robotics expansion risks driving avoidable growth in energy use, cost and associated emissions at the same time as the NHS is working toward net zero. The practical implication is therefore not that robotic surgery should be avoided, but that energy governance should be built into robotic service expansion from the outset.

Laparoscopy represents current ‘business-as-usual’ minimally invasive practice in many colorectal pathways and is the reference point for theatre sustainability improvements being pursued now through local Green Plans and professional guidance.^13^ Comparing robotics against laparoscopy therefore quantifies the additional electricity impact associated with the direction of travel (robotic scale-up), allowing sustainability measures to be designed in from the outset rather than retrofitted later.

Sustainability evaluation should not default to an anti-innovation position. Robotic surgery is forecast to expand; the priority is therefore to understand where robotics offers net value and how to reduce avoidable environmental burden as programmes scale. Several potential benefits may support robotic adoption in the right settings:

1. Access to minimally invasive surgery for complex cases: robotics may enable minimally invasive approaches for anatomically challenging procedures or patients who might otherwise undergo open surgery, with potential implications for recovery and resource use. In colorectal cancer surgery, favourable outcomes for robotic rectal cancer compared with laparoscopic approaches have been reported in large retrospective cohorts, although evidence varies by procedure and study design.^5^
2. Ergonomics and workforce sustainability: robotic platforms may improve surgeon ergonomics and reduce physical strain, which can support long-term workforce sustainability and productivity – an important ‘system sustainability’ outcome even if not captured in carbon accounting.^6,7^
3. Training and standardisation: structured training pathways and dual-console capability may support skill acquisition and more standardised operative workflows over time.^7,16^ Standardisation and learning curve gains may reduce operative duration and associated energy per case as teams mature.^3,7^
4. Technology evolution: future systems may improve size, usability and potentially operational efficiency, highlighting the value of establishing robust baseline measurements for comparison across platforms.^16,17^

Robotic surgery is frequently justified on clinical and pathway benefits such as reduced blood loss, lower conversion rates, or shorter length of stay. However, comparative evidence is heterogeneous and highly dependent on procedure type, case selection, team experience and local enhanced recovery pathways. While some studies report shorter length of stay with robotic colectomy in propensity-matched analyses,^18^ other comparisons do not demonstrate consistent differences across settings and procedure-specific evaluation remains essential. This variability strengthens the case for assessing sustainability at both the intraoperative level (electricity, consumables) and the whole-pathway level (bed-days, complications, readmissions) rather than assuming a uniform offset.

Although this study identifies higher electricity consumption associated with robotic equipment and longer time-in-theatre, the net environmental impact of adopting robotics at scale may depend on whole-pathway effects downstream of the operating room. In selected cohorts, robotics may reduce conversion to open surgery, complications or length of stay, which could offset some intraoperative energy penalties through reduced resource utilisation (e.g., bed-days, imaging, readmissions, critical care). Evidence regarding these outcomes is procedure-specific; for example, matched analyses in colectomy have reported reduced length of stay with robotics compared with other approaches.^18^ Accordingly, electricity measurements such as those presented here should be treated as a necessary operational baseline, to be integrated with future life cycle and pathway-level analyses when evaluating the net sustainability impact of robotic expansion.^10,13,19–21^

While electricity is directly measurable and operationally actionable, it does not capture the full carbon footprint. Published life-cycle assessment (LCA) literature in other specialties indicates higher environmental impacts for robotic procedures compared with laparoscopic comparators, including higher greenhouse gas emissions.^19,21^ Prospective multicentre work has also assessed climate impact comparisons between laparoscopic and robotic approaches.^20^ These findings support the argument that sustainability evaluation should extend beyond operational electricity to include consumables and waste, sterilisation/reprocessing, procurement, and supply chain emissions – particularly relevant given NHS net zero targets that include emissions the NHS can influence through purchasing.^8^

Given the projected increase in robotic surgery volume, reducing avoidable electricity demand should be incorporated into programme governance. The most actionable opportunities are likely to be avoidable standby use, unnecessary idle powered-on time, inefficient setup and clear-away workflows, and the absence of routine energy monitoring in robotic programme dashboards. RCS guidance outlines practical measures to improve sustainability in operating theatres, including waste reduction, green purchasing and operational changes that can be implemented without compromising care.^13^ An evidence review of ‘green theatre’ interventions further highlights achievable reductions, including switching off equipment when not in use.^10^ To translate these principles into robotic programme delivery, we propose the Energy Mitigation Bundle (Box 1), which defines minimum standards for measurement, standby governance, utilisation and procurement that can be embedded in programme dashboards and routine board reporting.

**Box 1:** Energy mitigation bundle for sustainable robotic surgery scale-up

Domain	Risk/driver	What to implement (minimum standard)	Metric/audit measure	Owner
Baseline measurement	Energy impact is invisible without routine measurement	Add energy to programme KPIs from day 1	kWh per case, kWh per theatre-hour, standby kWh/day, % cases with full shutdown of nonessential components	Robotic programme lead + Estates energy lead
Standby governance	Out-of-hours standby can materially contribute to annual energy use	Written ‘Robotics Power State SOP’ specifying: what must remain powered (manufacturer constraints) vs what must be powered down; clear responsibility and escalation	Weekly audit: overnight standby hours, weekend standby hours, compliance rate with SOP	Theatre coordinator + Robotics team + Estates
Power-state standardisation	Variable set-up practices increase idle powered-on time	Standardise power-up/power-down workflow; include a ‘power check’ in team brief/debrief	Idle powered-on minutes per list; time-to-ready and time-to-shutdown tracked for lists	Theatre team lead
Utilisation and workflow efficiency	Longer operative time increases kWh per case and overall theatre energy	Docking/undocking optimisation; instrument readiness; parallel processing (prep while system boots); ‘first-case on-time’ improvement	Docking time, turnaround time, list utilisation %, operative duration trend (learning curve)	Clinical lead + Theatre manager
Case selection strategy	Unselective use may increase energy without commensurate benefit	Define indications where robotics adds greatest clinical/system value (complex pelvic cases, difficult anatomy, conversion risk)	Quarterly review: robotics case-mix, conversion rate, complication rate, LOS, readmissions	MDT/Service line lead
Consumables and waste	Robotic consumables can increase waste volume and cost	Waste segregation and recycling pathways; review ‘single-use vs reusable where safe’; engage suppliers on packaging reduction	kg waste per case (if feasible), recycling rate, procurement waste initiatives logged	Theatre sustainability lead + Procurement
Procurement standards	Energy performance and standby requirements often absent from business cases	Add ‘energy and standby’ requirements to procurement specs and business cases (declared standby W, power-state options, service settings)	Procurement checklist compliance; supplier disclosure completeness	Procurement + Programme board
Estates and infrastructure alignment	Theatre HVAC dominates energy; robotics expansion must be planned with estates	Align robotics expansion with theatre ventilation optimisation, shutdown protocols, and Green Plan measures	Estates KPIs: theatre HVAC schedules, out-of-hours shutdown compliance	Estates lead
Reporting and governance	Without governance, improvements decay over time	Quarterly dashboard to programme board: energy KPIs + clinical outcomes + productivity	‘Robotics Sustainability Dashboard’ reviewed quarterly	Programme board chair

HVAC = heating, ventilation, and air conditioning; KPI = key performance indicator; LOS = length of stay; MDT = multidisciplinary team; SOP = standard operating procedure

## Limitations

This study measured electricity attributable to key laparoscopic/robotic equipment but did not quantify full pathway emissions (anaesthesia gases, disposables, sterilisation, supply chain, manufacturing/end-of-life). A full comparison would require LCA, building on published LCA literature.^19,21^ The study also used modelled setup, operative and clear-away durations rather than prospectively timing each individual case, and robotic and laparoscopic cases were not matched for case complexity. The results should therefore be interpreted as an equipment-energy comparison under realistic local workflow assumptions, rather than as a definitive case-matched comparison of all robotic and laparoscopic colorectal operations. Nevertheless, electricity is a material and modifiable contributor to theatre emissions and cost, and our results identify actionable opportunities – particularly out-of-hours standby.

## Conclusion

Robotic-assisted colorectal cancer surgery consumed over six times the electricity per case compared with laparoscopic surgery in our setting, with additional burden from out-of-hours standby. The main implication is that robotic equipment has higher electricity demand than laparoscopic equipment, and this difference may matter at scale even where the direct electricity cost per individual case is relatively modest. Given NHS England projections for rapid robotic scale-up and NHS net zero commitments (2040 to 2045), energy and carbon performance should be embedded into robotic programme design now.^8,11,12^ This includes routine energy measurement, standby management, theatre efficiency initiatives and procurement governance aligned to sustainable theatre guidance, so that increased robotic volume does not translate into avoidable increases in energy use and emissions.^10,13,14^

## Competing interests

The authors declare no competing interests.

## Funding

The authors received no financial support for the research, authorship and/or publication of this article.

## Ethics approval and consent to participate

No ethical considerations.

## Author contributions

**S Lodhia:** Conceptualisation, Formal analysis, Investigation, Writing – original draft. **V Pegna:** Data curation, Investigation, Methodology, Writing – review & editing. **T Rockall:** Conceptualisation, Supervision, Writing – review & editing.

## Artificial Intelligence

The authors declare that no AI was used to conduct the study or prepare the manuscript.
